# ICAM-1 Upregulation in Ethanol-Induced Fatty Murine Livers Promotes Injury and Sinusoidal Leukocyte Adherence after Transplantation

**DOI:** 10.1155/2012/480893

**Published:** 2012-06-18

**Authors:** Tom P. Theruvath, Venkat K. Ramshesh, Zhi Zhong, Robert T. Currin, Thomas Karrasch, John J. Lemasters

**Affiliations:** ^1^Center for Cell Death, Injury & Regeneration, Department of Pharmaceutical & Biomedical Sciences, Medical University of South Carolina, 280 Calhoun Street, MSC 140, Charleston, SC 29425, USA; ^2^Department of Surgery, Medical University of South Carolina, 280 Calhoun Street, MSC 140, Charleston, SC 29425, USA; ^3^Department of Cell & Developmental Biology, University of North Carolina, Chapel Hill, NC 27599, USA; ^4^Department of Medicine, University of Regensburg, 93053 Regensburg, Germany; ^5^Department of Biochemistry & Molecular Biology, Medical University of South Carolina, 280 Calhoun Street, MSC 140, Charleston, SC 29425, USA

## Abstract

*Background*. Transplantation of ethanol-induced steatotic livers causes increased graft injury. We hypothesized that upregulation of hepatic ICAM-1 after ethanol produces increased leukocyte adherence, resulting in increased generation of reactive oxygen species (ROS) and injury after liver transplantation (LT). *Methods*. C57BL/6 wildtype (WT) and ICAM-1 knockout (KO) mice were gavaged with ethanol (6 g/kg) or water. LT was then performed into WT recipients. Necrosis and apoptosis, 4-hydroxynonenal (4-HNE) immunostaining, and sinusoidal leukocyte movement by intravital microscopy were assessed. *Results*. Ethanol gavage of WT mice increased hepatic triglycerides 10-fold compared to water treatment (*P* < 0.05). ICAM-1 also increased, but ALT was normal. At 8 h after LT of WT grafts, ALT increased 2-fold more with ethanol than water treatment (*P* < 0.05). Compared to ethanol-treated WT grafts, ALT from ethanol-treated KO grafts was 78% less (*P* < 0.05). Apoptosis also decreased by 75% (*P* < 0.05), and 4-HNE staining after LT was also decreased in ethanol-treated KO grafts compared to WT. Intravital microscopy demonstrated a 2-fold decrease in leukocyte adhesion in KO grafts compared to WT grafts. Conclusions. Increased ICAM-1 expression in ethanol-treated fatty livers predisposes to leukocyte adherence after LT, which leads to a disturbed microcirculation, oxidative stress and graft injury.

## 1. Introduction

After cold ischemic liver storage for transplantation, reperfusion injury may lead to poor initial graft function and even graft failure. This injury is more severe and causes increased morbidity and mortality when steatotic donor livers are used [1, 2]. Because of the increasing incidence of nonalcoholic steatohepatitis in the general population and the association of vehicular accidents with steatosis-causing alcohol use and abuse, an important fraction of potential human donor livers is steatotic. Such marginal steatotic livers are increasingly used as liver grafts because of the liver donor shortage and the expanding waiting list for liver transplantation.

Sinusoidal endothelial cells and hepatocytes are particularly susceptible to ischemia/reperfusion (I/R) injury and consequent apoptotic and necrotic cell death, as shown by both *in vitro* and *in vivo* studies [3–6]. After liver I/R, recruitment of neutrophils and other inflammatory cells aggravates injury [7, 8]. Neutrophil recruitment also contributes to liver injury after endotoxin, sepsis, and chronic ethanol treatment [9–12]. Hepatic infiltration with neutrophils results in production of reactive oxygen species (ROS) and oxidative stress, resulting in neutrophil-mediated liver cell killing.

Intercellular adhesion molecule-1 (ICAM-1) is an endothelial- and leukocyte-associated transmembrane protein important in adherence of neutrophils to liver cells, including hepatocytes [13], and promotion of adherence-dependent oxidant stress, a major factor in neutrophil-mediated hepatocyte killing [10, 14]. However in a previous study, antibody blockade of ICAM-1, although decreasing white blood cell adherence, did not protect against I/R injury in a rat model of lean liver transplantation [15]. The importance of ICAM-1 in fatty liver transplantation has not been assessed. Accordingly, we compared liver injury in a murine model of fatty liver transplantation using wild-type and ICAM-deficient liver grafts. Our results show that ethanol treatment increases hepatic ICAM-1. Such ICAM-1 upregulation predisposes to leukocyte adherence, microcirculatory disturbances, oxidative stress, and increased graft injury after liver transplantation.

## 2. Materials and Methods

### 2.1. Ethanol Treatment and Donor Operation

All experiments were conducted using protocols approved by the Institutional Animal Care and Use Committee. Male C57BL/6 (wild-type) and ICAM-1-deficient mice, B6.129S4-*ICAM*1^*tm*1*Jcgr*^/*J* on a C57BL/6 background, were gavaged with 6 g/kg ethanol or water alone. Twelve hours after gavage, livers were harvested under ether anesthesia and stored in ice cold University of Wisconsin (UW), as previously described [16]. Time for the donor operation averaged 22 min.

### 2.2. Recipient Operation

 Livers from wild-type and ICAM-1-deficient mice were transplanted without rearterialization into wild-type mouse recipients under ether anesthesia, as previously described [16]. Both donor and recipient mice weighed 19–24 g. The recipient operation averaged 45 min, and portal vein clamp time averaged 15 min. For sham operations under ether anesthesia, wild-type and ICAM-1-deficient mice were laparotomized. After 45 min, the abdomen was closed.

### 2.3. Tissue Triglyceride Content

 Liver tissues (50 mg) of both wild type and ICAM-1 deficient mice were homogenized in water, and lipids were extracted into CHCl_3_ [17], dried in a vacuum centrifugal evaporator (Jouan RC 10.10, Thermo Scientific Inc., Atlanta, GA), and resuspended in 1 mL of CHCl_3_. An aliquot (50 *μ*L) was dried and resuspended in 100 *μ*L of isopropyl alcohol, 1% Triton X-100. Triacylglycerol (TAG) content was then determined using an enzymatic colorimetric method (Triglyceride Test Kit, Stanbio Laboratory, Boerne, TX).

### 2.4. Alanine Aminotransferase (ALT)

 Blood samples to measure ALT were collected from the inferior vena cava 8 h after transplantation for analysis by standard methods.

### 2.5. Histology

Histology was evaluated 8 h after liver transplantation. Liver tissues were fixed by immersion in 4% paraformaldehyde in phosphate-buffered saline and embedded in paraffin. Sections (4 *μ*m) were stained with hematoxylin and eosin (H&E). Ten random fields were assessed for necrosis by standard morphologic criteria (e.g., loss of architecture, vacuolization, karyolysis, increased eosinophilia). Images were captured on a microscope (Zeiss Axiovert 100 microscope, Thornwood, NY), and the area percentage of necrosis was quantified using a computer program (AxioQuant, BD Bioimaging Systems, San Jose, CA).

### 2.6. Cell Death Immunohistochemistry

Terminal deoxynucleotidyl transferase-mediated dUTP nick-end labeling (TUNEL) was performed on paraffin sections using an *in situ* cell death detection kit (Roche Diagnostics, Penzberg, Germany). TUNEL-positive parenchymal and nonparenchymal cells were counted by light microscopy in 10 random high-power fields (HPFs).

### 2.7. Lipid Peroxidation Immunohistochemistry

Lipid peroxidation was assessed immunocytochemically by detecting 4-hydroxy-2-nonenal (HNE) adducts with a rabbit 4-HNE antibody (Alpha Diagnostic International, San Antonio, TX) with visualization by anti-rabbit IgG horse radish peroxidase (HRP) and diaminobenzidine (DAB) chromogen according to the manufacturer's instructions (DAKO corporation, Carpinteria, CA). The slides were then counterstained with hematoxylin.

### 2.8. Intravital Imaging of White Blood Cell Adherence

At 4 h after transplantation, recipients were anesthetized with pentobarbital (50 mg/kg) and connected to a small animal ventilator via a tracheostomy and respiratory tube (20-gauge catheter), as previously described [16]. Briefly, a catheter (0.4 mm inner diameter, Zeus, Inc., Orangeburg, SC) was inserted into the right carotid artery. Using a syringe pump, rhodamine 6G (1 *μ*mol/mouse) was infused via the catheter over 20 min. During this time a laparotomy was performed using the previous incision line. After prone positioning of the mouse, the liver was gently withdrawn from the abdominal cavity and placed over a glass coverslip on the stage of a Zeiss Axiovert 100 microscope (Thornwood, NY). Images of rhodamine 6G fluorescence were collected with a 40X 1.2 NA water-immersion objective lens through a spinning disk confocal imaging attachment (Attofluor CARV Optical Module, BD Bioimaging Systems, San Jose, CA) to a 12-bit cooled CCD camera (Hamamatsu, Bridgewater, NJ). In 10 sec movies of 5 random fields per liver, white blood cells were scored for sticking (permanent adherence) and rolling (margination and slowing of white blood cell flow). Image analysis was performed in a blinded fashion using MetaFluor v.5.0 (Universal Imaging Corp., Downingtown, PA).

### 2.9. Statistical Analysis

Data are presented as means ± S.E., unless otherwise noted. Statistical analysis was performed by Student's *t*-test or ANOVA plus Student-Newman-Keuls test as appropriate, using *P* < 0.05 as the criterion of significance.

## 3. Results

### 3.1. ICAM-1 Upregulation in Ethanol-Induced Fatty Livers

**Figure 1:**
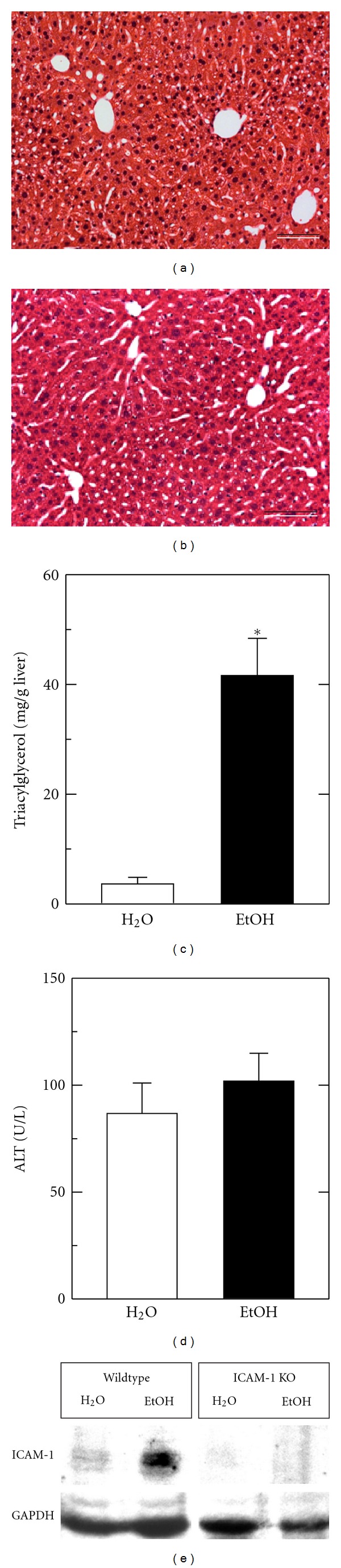
After ethanol gavage, mouse livers have increased fat content and ICAM-1 protein expression. Representative images of hepatic histology after ethanol gavage in wild type (a) and ICAM-1 deficient mice (b) are shown. Hepatic triacylglycerol (c), serum ALT (d), and hepatic ICAM-1 protein expression by Western blot (e) were assessed 12 h after water (H_2_O) and ethanol (EtOH) gavage, as described in Section 2. (e) shows upregulation of ICAM-1 after ethanol treatment and the absence of ICAM-1 in ICAM-1 deficient (KO) mice. Size of individual groups was 3-4. Bar is 50 *μ*m. *P < 0.05 versus H_2_O.

Wild type and ICAM-1 deficient mice were gavaged with ethanol or water, as described in Section 2. At 12 h after ethanol gavage, marked steatosis occurred to an equal extent in the livers of wild type and ICAM-1 deficient mice, which were indistinguishable histologically (compare Figures 1(a) and 1(b)). Overall, no differences in histology in livers of wild type and ICAM-1 deficient mice were observed either before or after ethanol treatment. After 12 h, serum ALT levels were normal and comparable in both wild type and ICAM-1 deficient mice (Figure 1(c)). However, triacylglycerol levels increased 10-fold after ethanol treatment compared to water treatment (*P* < 0.01, Figure 1(d)). Western blotting of homogenized liver tissue revealed increased ICAM-1 expression at 12 h after ethanol treatment and, as expected, absent ICAM-1 expression in ICAM-1 deficient livers (Figure 1(e)). Overall, although ICAM-1 upregulation did not occur in ICAM-1 deficient mice, hepatic histology, steatosis, and ALT release were comparable in wild type and ICAM-1 deficient mice after acute ethanol treatment.

### 3.2. Decreased ALT Release and Graft Necrosis after Transplantation of ICAM-1 Deficient Fatty Livers

**Figure 2:**
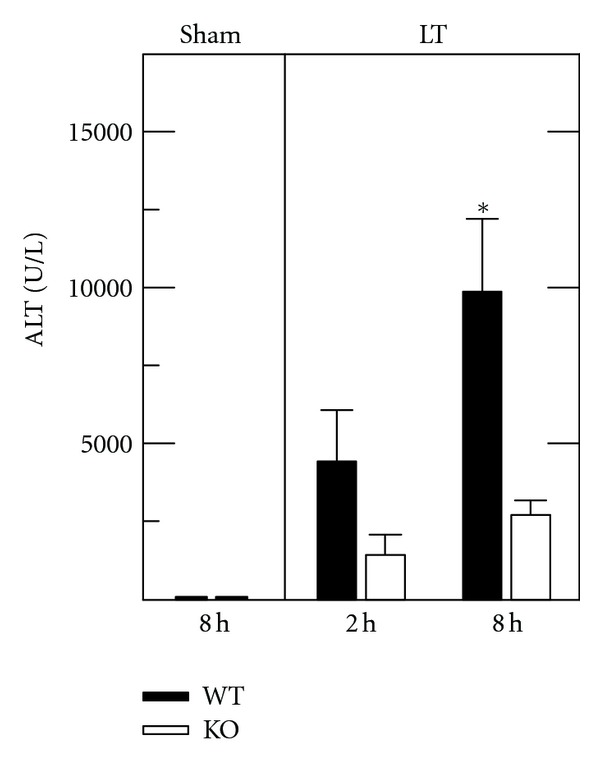
ALT release is decreased after transplantation of ICAM-1 deficient fatty livers. Serum ALT was assessed 8 h after sham operation (Sham) and 2 and 8 h after mouse liver transplantation (LT), as described in Section 2. Transplants were performed into WT recipients after ethanol treatment of wild type (WT) or ICAM-1 deficient (KO) liver donors. Sham groups had normal and comparable values to unoperated animals, and only sham is shown. Group sizes were sham WT and KO, 3; LT WT, 5; LT KO, 6. *P < 0.05 versus KO grafts.

At 8 h after sham operation in ethanol-treated animals, wild type and ICAM-1 deficient mice had normal and comparable serum ALT averaging 118 ± 13 U/L (Figure 2). In contrast, ALT increased markedly in recipient mice after ethanol treatment, 12 h storage, and transplantation of livers. At 2 h after transplantation, ALT increased to 4,431 ± 1,636 U/L and 1,423 ± 656 U/L (*P* = 0.1), respectively, in recipients of wild type and ICAM-1 deficient livers. At 8 h after transplantation, ALT increased to 9,870 ± 2,344 U/L and 2709 ± 458 U/L, respectively (*P* < 0.05) (Figure 2). Thus, at 8 h following transplantation, ALT was 72% less in ICAM knockout than wild type liver recipients.

**Figure 3:**
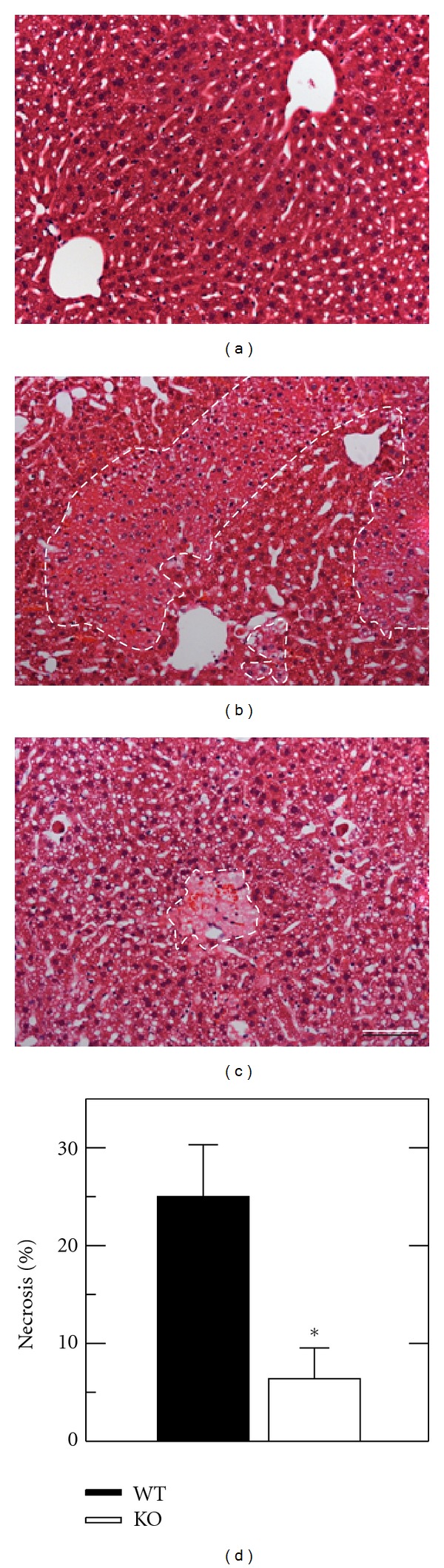
Necrosis is decreased after transplantation of ICAM-1 deficient fatty livers. Mouse livers were transplanted, as described in Section 2. At 8 h postoperatively, necrosis was assessed by H&E histology after sham operation (a), transplantation of wild type (WT) livers (b), and transplantation of ICAM-1 deficient (KO) livers (c). (d) shows necrosis as percent area in liver sections averaged from 5 livers per group. Necrosis in sham-operated WT and KO livers was absent. Bar is 50 *μ*m. *P < 0.05 versus WT grafts.

Graft injury was also assessed histologically. Liver histology was normal and indistinguishable in wild type and ICAM-1 deficient mice with and without sham operation (Figure 3(a) and data not shown). At 8 h after transplantation of wild type livers, large areas of necrosis were present with a predominantly pericentral and midzonal distribution (Figure 3(b)). By comparison, necrosis was decreased after transplantation of ICAM-1 deficient livers (Figure 3(c)). Morphometry revealed a decrease of hepatic necrosis from 25 ± 5.3% after wild type liver transplantation to 6.5 ± 3.1% after transplantation of ICAM-1 deficient livers (*P* < 0.05) (Figure 3(d)). Thus, transplantation of ICAM-1 deficient fatty livers decreased hepatic necrosis by three quarters.

### 3.3. Decreased Graft Apoptosis after Transplantation of ICAM-1 Deficient Fatty Livers

**Figure 4:**
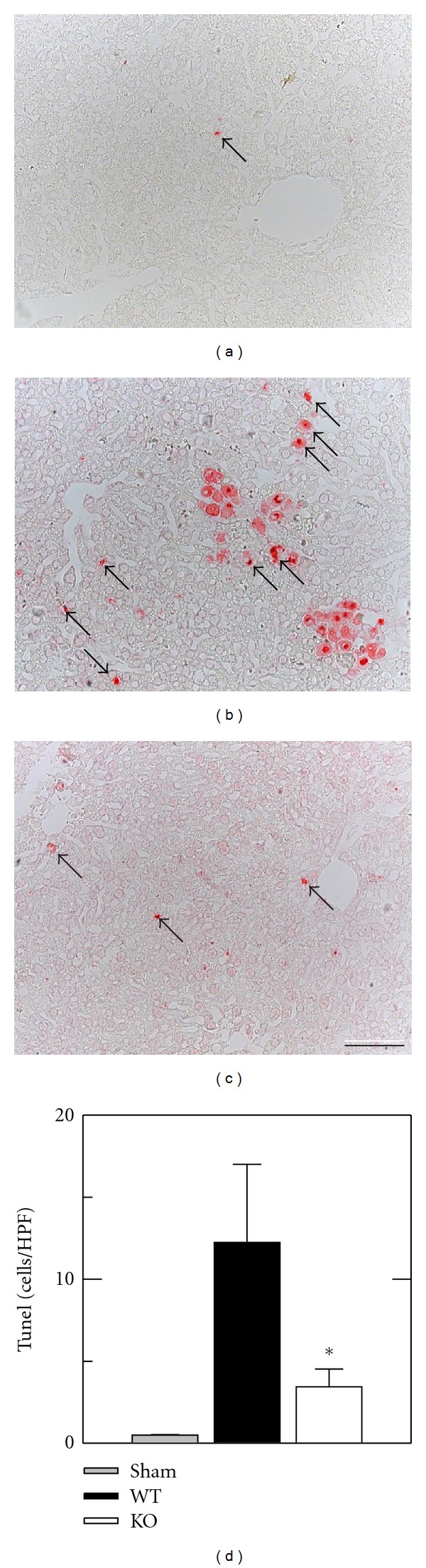
Apoptosis is decreased after transplantation of ICAM-1 deficient fatty livers. Mouse livers were transplanted, as described in Section 2. At 8 h postoperatively, TUNEL was assessed in tissue sections after sham operation (a), transplantation of wild type (WT) livers (b), and transplantation of ICAM-1 deficient (KO) livers (c). (d) quantifies the TUNEL-positive cells per high power field (HPF). TUNEL for WT sham was virtually zero and comparable to KO sham, and only KO sham is plotted. Bar is 50 *μ*m. *P < 0.05 versus WT grafts.

TUNEL was performed on tissue sections to assess double-stranded DNA breaks that are characteristic of apoptosis. TUNEL-positive cells were rare in wild type and ICAM-1 livers with and without sham operation, averaging less than 1 cell/HPF (Figures 4(a) and 4(d), and data not shown). At 8 h after transplantation with wild type livers, TUNEL in nonnecrotic areas increased to 12.2 ± 4.8 cells/HPF without apparent zonal localization (Figures 4(b) and 4(d)). After transplantation of ICAM-1 deficient livers, TUNEL decreased by about two-thirds to 3.5 ± 1.1 cells/HPF (*P* < 0.05, Figures 4(c) and 4(d)). As a percentage of all cells, TUNEL was 2.6 ± 1.3% after transplantation of wild type livers versus 0.7 ± 0.3% after transplantation of ICAM-1 deficient livers (*P* < 0.05).

### 3.4. Decreased White Blood Cell Adhesion in Fatty ICAM-1 Deficient Liver Grafts

**Figure 5:**
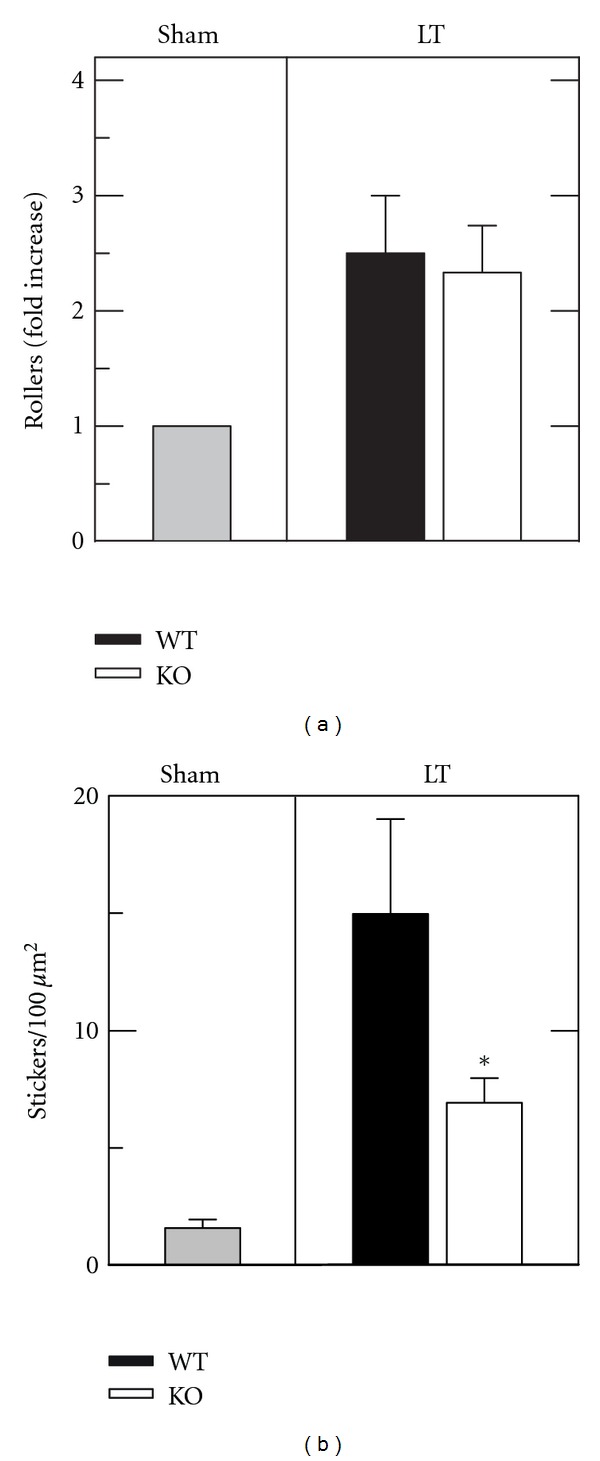
ICAM-1 deficient grafts show decreased leukocyte adherence but unchanged rolling margination after ethanol-induced fatty mouse liver transplantation. Livers were transplanted (LT) or subjected to sham operation (Sham) and visualized by intravital confocal microscopy of rhodamine 6G fluorescence after 4 h, as described in Section 2. Rhodamine 6G-fluorescing leukocytes were scored for slow flow rolling margination (Rollers, (a)) and no flow adherence to sinusoidal walls (Stickers, (b)) as either fold increase versus sham (a) or as absolute number per *μ*m^2^(b). WT and KO sham groups were comparable, and only the KO sham group is plotted. Individual group size was 4. *P < 0.05 versus WT grafts.

At 4 h after sham operation, intravital confocal microscopy revealed bright fluorescence of rhodamine 6G-labeled white blood cells moving through hepatic sinusoids. No differences were seen in wild type or ICAM-1 deficient mice after sham operation and only occasional margination (rolling) and sticking of rhodamine 6G-labeled cells were noted in sinusoids (Figures 5(a) and 5(b), and Video A of supplemental data in Supplementary Material available online at doi:10.11/2012/480893). By contrast, at 4 h after liver transplantation of fatty wild type livers, marginating (rolling) and adherent (stickers) rhodamine 6G-stained cells increased markedly (Figure 5, Video B of supplemental data).

After transplantation of ICAM-1 deficient livers, fewer nonmobile rhodamine 6G-stained cells (stickers) were present in hepatic sinusoids (Video C of supplemental data). Rhodamine 6G-stained rollers and stickers were scored and counted for each liver. In ethanol-treated sham-operated livers, 1.6 ± 0.36 stickers/100 *μ*m^2^ were identified. After transplantation of fatty WT livers, stickers increased to 15.0 ± 4.04/100 *μ*m^2^, a more than 8-fold increase. Similarly, rollers increased by 2.5-fold after wild type transplantation compared to sham (Figures 5(a) and 5(b)). After transplantation of ICAM-1 deficient livers, stickers decreased 54% to 6.9 ± 1.04 per 100 *μ*m^2^ (*P* < 0.05 versus wild type) (Figure 5(b)). By contrast, rollers did not decrease in ICAM-1 compared to wild type liver grafts (Figure 5(a)). Thus, sinusoidal adherence (sticking) but not initial margination (rolling) of white blood cells was decreased in ICAM-1 deficient compared to wild type grafts after liver transplantation.

### 3.5. Decreased Oxidative Stress after Transplantation of Fatty ICAM-1 Deficient Livers

**Figure 6:**
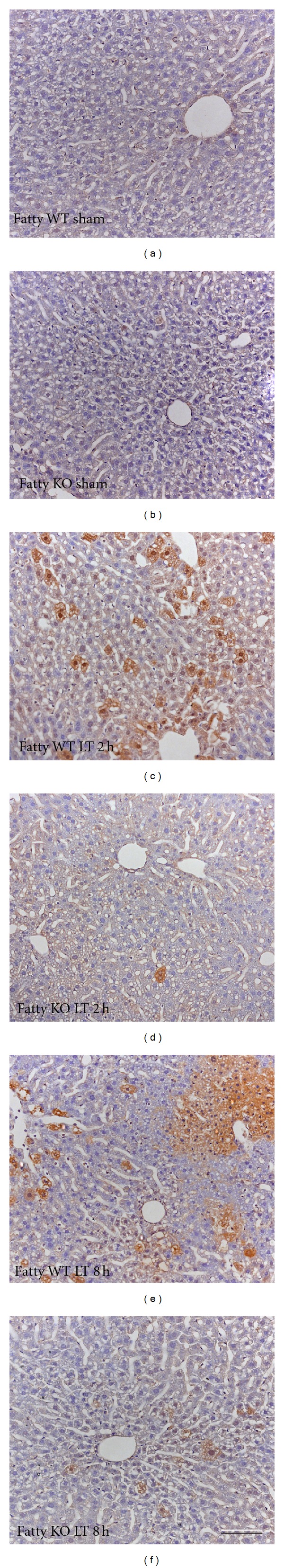
Decreased lipid peroxidation occurs in ICAM-1 deficient liver grafts. At 2 and 8 h after transplantation or sham operation, immunohistochemical staining was performed for 4-hydroxynonenal-modified proteins (4-HNE, *brown*), as described in Section 2. Panels are sham-operated wild type (WT) livers (a), sham-operated ICAM-1 deficient (KO) livers (b), WT liver grafts at 2 h after reperfusion (c), KO liver grafts at 2 h after reperfusion (d), WT liver grafts at 8 h after reperfusion (e), and KO liver grafts at 8 h after reperfusion (f). Brown HNE immunoreactivity was present in WT grafts after 2 and 8 h ((c) and (e)), which was markedly decreased in KO livers ((d) and (f)). Size of groups was 4. Bar is 50 *μ*m.

We used 4-HNE immunocytochemistry as a marker of oxidative stress in liver grafts at 2 h and 8 h after reperfusion. HNE adduct formation is a consequence of lipid peroxidation resulting from ROS generation. In livers of ethanol-treated wild type and ICAM-1 deficient mice at 8 h after sham operation, HNE brown staining was nearly undetectable (Figures 6(a) and 6(b)). By contrast, at 2 h after transplantation of fatty wild type livers, HNE staining in the cytoplasm and nuclei of hepatocytes was present in a mosaic pattern throughout the tissue sections (Figure 6(c)). After 8 h, HNE staining became confluent in midzonal and pericentral areas with sparing of periportal areas (Figure 6(e)).

Compared to wild type, far fewer hepatocytes of fatty ICAM-1 deficient liver grafts stained for HNE both at 2 h and 8 h after reperfusion (Figures 6(d) and 6(f)). Thus, oxidative stress in ICAM-1 deficient fatty liver grafts was decreased compared to wild type fatty grafts.

## 4. Discussion

The limiting factor in clinical liver transplantation is donor shortage, which leads to death of patients on the waiting list. Expanding the donor pool by including marginal steatotic donor livers would help shorten wait times and increase the availability of donor livers for transplantation. However, such increased use requires overcoming the increased susceptibility of fatty liver grafts to poor initial function and failure. Targeting specific pathways to decrease reperfusion injury of cold stored steatotic livers might thus be a beneficial approach to improve the function and survival of fatty liver grafts.

The aim of the present study was to evaluate the importance of ICAM-1 in graft injury after transplantation of ethanol-induced steatotic mouse livers. ICAM-1 has previously been shown to contribute to hepatic injury after various nonsurgical liver stresses [18, 19] and ICAM-1 blockade demonstrated less white blood cell adherence after lean rat liver transplantation [15]. We tested the hypothesis that ICAM-1 upregulation in ethanol-induced fatty livers leads to necrosis and apoptosis after transplantation through sinusoidal leukocyte adherence and subsequent ROS generation. We evaluated graft injury by enzyme release, necrosis and apoptosis, white blood cell adherence by intravital microscopy, and oxidative stress by HNE immunocytochemistry after transplantation. Our findings demonstrate that ethanol treatment upregulates ICAM-1 expression and that ICAM-1 deficiency decreases injury, leukocyte adherence and oxidative stress in ethanol-induced steatotic liver grafts. Our results are consistent with the conclusion that protection in ICAM-1 deficient grafts is the consequence of decreased sinusoidal adherence of white blood cells and decreased ROS formation.

Previous studies utilizing rodents demonstrate that doses of 5-6 g/kg ethanol increase hepatic triglyceride and decrease graft survival after transplantation substantially [20, 21]. Such a dose produces a peak blood ethanol concentration of about 370 mg/dL after 2 h in rats, which declines to undetectable levels in 8 to 10 h. No respiratory suppression is observed in these ethanol-treated animals receiving this treatment. In humans, fatty liver occurs to a similar extent after an acute ethanol binge [22]. Accordingly, rodent models have been used for decades to investigate the extent of ethanol-induced liver steatosis in a variety of contexts. In our experimental setting in mice, a single high dose of ethanol (6 g/kg) administered by gavage produced prominent hepatic steatosis 12 h later and a 10-fold increase of hepatic triacylglycerol content (Figure 1). Steatosis was associated with increased hepatic ICAM-1 expression (Figure 1(e)). Although blood alcohol peaked at about 580 mg/dL at 40 min after this treatment (data not shown), increases of necrosis, apoptosis, and serum ALT were negligible, and mortality did not occur. Interestingly, ethanol-induced upregulation of ICAM-1 alone did not increase white blood cell adherence before cold storage and reperfusion (Figure 5 and Video A of supplemental data). However, after transplantation of ethanol-induced fatty livers, white blood cell adherence increased markedly, an effect attenuated by more than half in ICAM-1 deficient liver grafts (Figure 5 and Videos B and C of supplemental data). Injury was also decreased in ICAM-1 deficient grafts (Figures 2, 3, and 4).

Previously in a study of warm liver I/R, antibody against ICAM-1 did not improve outcome when used alone but only when combined with antibodies against lymphocyte-function-associated antigen-1 (LFA-1) and beta 2 integrin, (CD-18), which are binding partners for ICAM-1 present on leukocyte membrane surfaces [23]. Similarly, after lean liver transplantation, ICAM-1 antibodies failed to prevent graft injury, although leukocyte adherence was decreased [15]. Thus, ICAM-1-dependent leukocyte adherence may itself be insufficient to cause liver injury and instead may act synergistically with other alterations to cause tissue damage. In the setting of transplantation of ethanol-induced fatty livers, our studies would suggest that ethanol-induced sinusoidal ICAM-1 upregulation is a first hit that alone is insufficient to cause hepatic injury. After transplantation, however, activation of leukocytes may represent a second hit, which when combined with the first hit causes hepatic necrosis, apoptosis, and enzyme release. ICAM-1 in our setting of fatty liver transplantation had more impact on injury than in previous models of warm hepatic I/R and lean liver transplantation. Ethanol-induced upregulation of ICAM-1 prior to I/R or transplantation stress may be particularly important in predisposing livers to injury, and ICAM-1 upregulation could potentially represent a biomarker to susceptibility of fatty livers to storage/reperfusion injury in human clinical transplantation.

Although leukocyte adherence (sticking) was decreased in ICAM-1 deficient liver grafts, rolling margination of leukocytes was unchanged (Figure 5, Videos B and C in supplemental data). These observations are consistent with earlier studies showing that endothelial selectins mediate rolling margination of leukocytes in response to chemokines and other proinflammatory signals, whereas ICAM-1 mediates adherence and infiltration into tissue [24].

ROS production by ischemic tissue after reperfusion is widely held as a major factor contributing to I/R injury. ROS generation also contributes to cold storage/reperfusion injury [21, 25–32]. ROS induces tissue damage by activating mitochondrial pathways leading to necrosis and apoptosis and through direct attack on proteins, lipids, and DNA. HNE is a product of ROS-dependent peroxidation of *ω*-6 polyunsaturated fatty acids, such as linoleic and arachidonic acid [33]. HNE reacts with protein sulfhydryls to form covalent adducts that can be detected by immunocytochemistry. In the present study, HNE adducts developed after wild type liver transplantation that were markedly decreased in ICAM-1 deficient liver grafts (Figure 6). Even at 2 hours after transplantation before onset of necrosis, HNE adducts were increased in wild type grafts but markedly decreased in ICAM-1 deficient grafts (Figure 6). These findings indicate that ROS production is temporally upstream of necrosis, which does not occur maximally until 4 or more hours after transplantation (Figures 2 and 3 and data not shown, see also [25, 26, 34–36]). Thus, ICAM-1-dependent leukocyte margination likely contributes to ROS generation and graft injury after wild type liver transplantation.

In summary, our results demonstrate involvement of ICAM-1 in storage/reperfusion injury to ethanol-induced fatty liver grafts. Prior to transplantation, ethanol treatment causes steatosis and upregulation of ICAM-1 expression in donor wild type livers. Such ICAM-1 upregulation was associated with increased leukocyte adherence, ROS generation, and injury to liver grafts. Thus, ICAM-1 upregulation and signaling in fatty liver grafts could represent a biomarker and target to identify and decrease susceptibility to storage/reperfusion injury of fatty liver grafts. However, the effects of chronic alcohol exposure or other means of fatty liver induction on graft injury, oxidative stress, and leukocyte recruitment may be different from those observed after acute ethanol administration. Thus, future studies will be needed to determine what benefit, if any, ICAM-1 targeting might have on fatty liver grafts in human clinical liver transplantation.

## Supplementary Material

Intravital Video Imaging. ICAM-1 deficient grafts show decreased leukocyte adherence but unchanged rolling margination after ethanol-induced fatty mouse liver transplantation. Livers were transplanted (LT) or subjected to sham operation (Sham) and visualized by intravital confocal microscopy of rhodamine 6G fluorescence at 4 h after surgery. Following prone positioning of the mouse, the liver was gently withdrawn from the abdominal cavity and placed over a glass coverslip on the stage of a Zeiss Axiovert 100 microscope (Thornwood, NY). Images of rhodamine 6G fluorescence were collected with a 40X magnification, 1.2 NA water-immersion objective lens through a spinning disk confocal imaging attachment (Attofluor CARV Optical Module, BD Bioimaging Systems, San Jose, CA) to a 12 bit cooled CCD camera (Hamamatsu, Bridgewater, NJ). In 10 sec movies of 5 random fields per liver, white blood cells were scored for sticking (permanent adherence) and rolling (margination and slowing of white blood cell flow). Size of groups was 4-5.
Video A: This video demonstrates an ethanol treated sham liver without noticeable rolling margination (rollers) and no flow adherence to sinusoidal walls (stickers).
Video B: This sequence demonstrates an ethanol-treated fatty WT liver graft with noticeable stickers and increased number of rolling rhodamine 6G positive cells.
Video C: This video shows an ethanol-treated fatty ICAM-1 KO liver graft with less adherent rhodamine 6G positive cells.

## Abbreviations

ALT: Alanine aminotransferase
ATP: Adenosine triphosphate
CD-18: Beta 2 integrin
DAB: 3,3′-diaminobenzidine
H&E: Hematoxylin and eosin
HNE: 4-hydroxynonenal
HPF: High power field
HRP: Horse radish peroxidase
ICAM-1: Intercellular adhesion molecule-1
I/R: Ischemia/reperfusion
LFA-1: lymphocyte function-associated antigen-1
LT: Liver transplantation
ROS: Reactive oxygen species
TUNEL: Terminal deoxynucleotidyl transferase-mediated dUTP nick-end labeling
UW: University of Wisconsin cold storage solution.
